# Strength training in the gym versus specific strength training on the bike in young off-road cyclists

**DOI:** 10.1093/bmb/ldaf024

**Published:** 2026-01-03

**Authors:** Domenico Savio Salvatore Vicari, Luca Filipas, Valerio Giustino, Patrik Drid, Antonino Bianco

**Affiliations:** Department of Neurosciences, Biomedicine and Movement Sciences, University of Verona, Via Casorati 43, 37131, Verona, Italy; Sport and Exercise Sciences Research Unit, Department of Psychology, Educational Science and Human Movement, University of Palermo, Via Giovanni Pascoli 6, 90144, Palermo, Italy; Department of Biomedical Sciences for Health, University of Milan, Via Giuseppe Colombo 71, 20133, Milan, Italy; TotalEnergies Pro Cycling Team, 19 Rue DU Dr Arsene Mignen, 85140, Essarts-en-Bocage, France; Sport and Exercise Sciences Research Unit, Department of Psychology, Educational Science and Human Movement, University of Palermo, Via Giovanni Pascoli 6, 90144, Palermo, Italy; Faculty of Sport and Physical Education, University of Novi Sad, Lovćenska 16, 21101, Novi Sad, Serbia; Sport and Exercise Sciences Research Unit, Department of Psychology, Educational Science and Human Movement, University of Palermo, Via Giovanni Pascoli 6, 90144, Palermo, Italy

**Keywords:** cycling, bicycle, resistance training, sport performance, biomechanics

## Abstract

**Background:**

The benefits of strength training in cyclists are still a topic of debate. The aim of this study was to compare the effects of strength training performed in the gym with specific strength training performed on the bike in young off-road cyclists.

**Sources of data:**

Nineteen participants were divided into the following two groups: (i) the group A performed 12 weeks of endurance training combined with two sessions/week of strength training in the gym; (ii) the group B performed 12 weeks of endurance training combined with two sessions/week of specific strength training on the bike. Incremental test, 30 s Wingate test, and countermovement jump test were conducted at pre (T0) and post (T1) training programs.

**Areas of agreement:**

The findings are in line with previous research showing that heavy strength training in cyclists has significant improvements in some aerobic and anaerobic performance outcomes. Indeed, our results showed that in the group A, the Mean Power Output (MPO) of the 30 s Wingate test increased significantly between T0 and T1 (*P* = .002). In the group B, no significant differences were found in the MPO of the 30 s Wingate test between T0 and T1 (*P* = .276). In the group A, the Maximum Power Achieved (MPA) increased significantly between T0 and T1 (*P* < .001). In the group B, no significant differences were found in the MPA between T0 and T1 (*P* = .889).

**Areas of controversy:**

Strength training in the gym for cyclists has not been widely investigated, and, in fact, the literature shows conflicting results on the topic, with some research groups highlighting the importance of sport-specific strength training. In this way, in both groups, no significant differences in the vertical jump height between T0 and T1 (*P* = .331 and *P* = .184, for groups A and B, respectively) were detected. However, the small sample size and the numerical heterogeneity between males and females in the recruited sample do not allow to generalize findings.

**Growing points:**

As the group of cyclists who performed strength training in the gym showed significant improvements, this study suggests integrating heavy strength sessions in the gym into the usual endurance training program.

**Areas timely for developing research:**

Further studies should investigate the effectiveness of strength training in the gym both in all off-road cycling categories as well as in other cycling specialities.

## Introduction

The training of metabolic capacities in off-road cycling is critical, given the performance model of the competition [1–3]. The duration of cross-country races depends on the type of category. For elite cyclists, the duration of the race is ~120/135 minutes for men and 105/120 minutes for women [1]. For cyclists in the Italian youth categories “Esordienti” and “Allievi,” the duration of these races is ~30 and 45 minutes, respectively. The races are held at high intensity with a heart rate of ~90% of maximum, which corresponds to 84% of VO2max [3]. These physical demands underscore the importance of aerobic metabolism in this sport. The cross-country races involve explosive mass starts and numerous laps on an off-road circuit with different climbs and descents featuring gravel, rock gardens, and roots [3]. The explosive mass starts and very steep climbs require very high power outputs (up to 500 W) [1]. The anaerobic metabolism also appears to be a determinant factor for performance in off-road cycling and, therefore, deserves attention in training planning.

Since neuromuscular capacities can influence athletes’ performance [4], strength training, which is very often underestimated, can play a key role in off-road cyclists’ performance. There are still no clear guidelines in the scientific literature on strength training in cyclists. For this reason, there are cyclists who prefer to train strength on the bicycle, and other athletes who train strength in the gym using overloads [5,6].

On the bicycle, there are several specific exercises to stimulate strength development that can be performed at high intensity with low pedaling cadences. This training involves sessions often performed uphill at high intensity with a pedaling cadence of around 40–70 rpm [7]. Pedaling at low cadences allows for a higher expression of force for each pedaling cycle. This is because power is given by the force expressed per pedal strokes per minute, so for the same power, more force will be expressed at a lower pedaling cadence [7].

With regard to strength training in the gym, there are different training methods widely investigated in the literature, such as the velocity-based training (VBT) and the percent-based training (PBT) [4]. In the VBT, the speed of movement of an exercise is measured with a linear position transducer and used to measure the intensity of the exercise [8–10]. On the other hand, the PBT involves measuring the intensity of the exercise as percentage of the one maximum repetition (%1RM). Most often, strength training in the gym in cycling is done during the winter, that is, in the general preparation period in which cyclists do not have races. This is also because strength training may require long recovery times. Moreover, during the competition season, strength training in the gym is more difficult because elite cyclists are often in training camps and do not have facilities available. It is a different matter for cyclists of youth categories who could train constantly in the gym to develop strength, laying the foundations for a more solid preparation. There are studies in the literature that have analyzed the effects of strength training in the gym on cycling performance such as power output [5,6,11,12], although this practice is not common among coaches for the misconception that strength training in the gym could be disadvantageous since it could lead to an increase in body mass which would negatively affect power-to-weight ratio, resulting in worsening performance [13]. However, strength training in the gym planned with the goal of increasing functional strength for cycling should not cause concern. Indeed, an increase in strength should compensate for any additional increase in body mass. In the gym, the most common exercises used to stimulate strength in cyclists are those with closed kinetic chain and that adopt a knee joint flexion of <90 degrees, reproducing the pedaling movement.

It is well known the benefits of strength training on endurance performance [13–16], as well as on injury prevention [17]. However, considering the limited literature on the topic, it is still unclear whether strength training in the gym or specific strength exercises at low pedaling cadences on the bike is the best in terms of strength development.

To the best of our knowledge, to date, there are no studies that have investigated the effects of strength training in young off-road cyclists.

The aim of this study was to compare the effects of strength training performed in the gym with specific strength training performed on the bike in young off-road cyclists. Specifically, we compared the effects of 12 weeks of endurance training combined with two sessions per week of strength training in the gym, with 12 weeks of endurance training combined with two sessions per week of specific strength training on the bike. The hypothesis is that the group of cyclists who performed endurance training combined with strength training in the gym could have higher improvements in power output (Wingate and incremental tests) and vertical jump height (countermovement jump test), compared to the group of cyclists who performed endurance training combined with specific strength training on the bike at low pedaling cadences.

## Methods

### Experimental design

This longitudinal study was conducted during the winter season, the period of general preparation. All participants were recruited through the Research and Development Technical Centre of the Sicilian Regional Committee of the Italian Cycling Federation. By using the random number generation function in Microsoft Excel (Microsoft Corporation; Redmond, WA, USA) as randomization procedure, the recruited participants were randomly divided into the following two groups: (i) the group A performed 12 weeks of endurance training combined with two sessions per week of strength training in the gym; (ii) the group B performed 12 weeks of endurance training combined with two sessions per week of specific strength training on the bike using low pedaling cadences.

The tests consisted of an incremental test, 30 s Wingate test, and a countermovement jump test, which were conducted at pre (T0) and post (T1) training programs. The characteristics of the two groups are reported in Table 1.

**Table 1:** Characteristics of the two groups

	Group A	Group B
N	11 (8 male, 3 female)	8 (8 male)
Age (years)	14.1 ± 1	14.1 ± 1.3
Weight (kg)	49 ± 10	52.5 ± 12.2
Height (cm)	159 ± 7.9	163 ± 11.8

Group A, cyclists who performed strength training in the gym; Group B, cyclists who performed strength training on the bike.

The study was conducted in accordance with the recommendations of the Declaration of Helsinki for the involvement of people in research and was approved by the Bioethics Committee of the University of Palermo (n. 217/2024).

### Participants

Nineteen young off-road cyclists (16 m, 3f), aged between 13 and 16 years old and belonging to the youth categories “Esordienti” and “Allievi” of the Italian Cycling Federation, were recruited for this study. For participation to the study, the young off-road cyclists had to have a cycling practice of at least two years and had to train 6 days per week. Cyclists who had suffered injuries in the 6 months prior to study recruitment were excluded. All participants concluded the experimental period without stopping.

Since participants were minors, parents provided an informed written consent for participation of their children in the study.

### Endurance training

Both groups performed the training program 6 days per week for 12 weeks. In detail, 3 days per week they performed a two-hour endurance training session (at an intensity between 73% and 82% of their maximum heart rate calculated with an incremental test at T0), 2 days per week they performed a strength training (in the gym and on the bike for the group A and the group B, respectively), 1 day per week they trained their downhill and uphill riding skills with their off-road bike, 1 day per week they rested.

To track the endurance training intensity, their heart rates were constantly monitored. After each training session, the recorded heart rate data was uploaded to a training platform (TrainingPeaks, 285 Century Pl, Louisville, CO 80027).

Table 2 shows the structure of the training during the week.

**Table 2:** Structure of the training during the week

	Monday	Tuesday	Wednesday	Thursday	Friday	Saturday	Sunday
Group A	Endurance training	Strength training in the gym	Endurance training	Mtb skills training	Strength training in the gym	Day-off	Endurance training
Group B	Endurance training	Strength training on the bike	Endurance training	Mtb skills training	Strength training on the bike	Day-off	Endurance training

Group A, cyclists who performed strength training in the gym; Group B, cyclists who performed strength training on the bike.

### Strength training in the gym

The strength training in the gym was performed twice a week for a total of 24 sessions. Each session consisted of 15 minutes of warm-up on a spinning bike, 10 minutes of joint mobility exercises, a central part including strength exercises, and 10 minutes of cool-down on a spinning bike.

Strength exercises were half squats, deadlifts from a standing position, dumbbell lunges, and Bulgarian squats performed with an increasing intensity and volume over the weeks, with a recovery of 2.5 minutes between series and 5 minutes between exercises.

Considering that it has been shown that the peak force during pedaling occurs at ~100 degrees of knee flexion, the exercises were performed up to 90 degrees of knee flexion [18,19]. The exercises were performed within 1 s in the concentric phase and 2–3 s in the eccentric phase [12,19]. The training sessions were supervised by a coach who, at the same time, recorded all the training loads in a diary. Table 3 shows the structure of the strength training in the gym for the group A.

**Table 3:** Structure of the strength training program in the gym for the group A

Week	1	2	3	4	5	6
Load (% of 1RM)	65/70	65/70	65/70	70/75	70/75	70/75
Repetitions	12	12	12	8	8	8
Set	4	4	4	3	3	3
Week	7	8	9	10	11	12
Load (% of 1RM)	75/80	75/80	75/80	80/85	80/85	80/85
Repetitions	6	6	6	4	4	4
Set	2	2	2	2	2	2

Group A, cyclists who performed strength training in the gym.

### Strength training on the bike

The strength training on the bike was performed twice a week for a total of 24 sessions. The exercises for the specific strength training on the bike consisted of maximum sprints from a standstill with a constrained gear performed on their bike (52/18 with 6.20 meters of metric development for “Esordienti” and 52/16 with 6.94 meters of metric development for “Allievi”) for a duration of 12 s, in which the pedaling cadence went from 0 to 90 rpm. There was a recovery of 2.5 minutes between reps and 10 minutes between sets at self-selected pedaling intensity and cadence.

This training was performed on a climb with 4/5% of slopes under the supervision of a coach. If an athlete with a constrained gear failed to stay within the pedaling cadence range, the coach would change gears so that the cyclist would be within the required pedaling cadence range. During these sprints, cyclists were asked to remain seated on the saddle and push as hard as they could. Table 4 shows the structure of the strength training on the bike for the group B.

**Table 4:** Structure of the strength training program on the bike for the group B

Week	1	2	3	4	5	6
Repetitions	6	6	6	8	8	8
Set	3	3	3	3	3	3
Week	7	8	9	10	11	12
Repetitions	6	6	6	8	8	8
Set	4	4	4	4	4	4

Group B, cyclists who performed strength training on the bike.

### Testing

Two test days were performed 48 hours apart, both at pre (T0) and post (T1) training programs, following a block order.

On the first test day, each participant was asked to warm-up for 5 minutes by performing body weight squat jumps, and afterwards, the countermovement jump test was administered. After 1 hour, each participant was asked to warm-up on their bike for 10 minutes at a self-selected pedaling intensity and cadence, and afterwards, the 30-s Wingate test was administered. After 5 minutes, the incremental test was administered. Since pedaling kinematics could influence test performance [20], all participants underwent a bike fitting procedure using a 3D kinematic analysis system (STT Systems; San Sebastian, Spain) and a saddle pressure analysis system (W-Saddle Pro, LetSense Group; Castel Maggiore, Bologna, Italy), focusing on the sex of the participants [21]. Both the 30-s Wingate test and the incremental test are the most common for evaluating cyclists’ performance [22].

On the second test day, the 1RM of the squat, dumbbell lunges, deadlift, and Bulgarian squat were assessed after a 10-minute period of free warm-up and joint mobility supervised by a coach.

As testing was repeated at T1, it was carried out on the same day and at the same time as at T0 and in the same block order. The same researcher conducted the testing procedure both at T0 and T1.

### Wingate test

The 30-s Wingate test was performed by each participant with their bike installed on a specific roller (MagneticDays; Foiano della Chiana, Arezzo, Italy). Each participant was asked to warm-up for 10 minutes at a self-selected pedaling intensity and cadence. The test began after each participant had rested for one minute. Three seconds before the start of the test, participants were asked to pedal at a low cadence (~65 rpm) with the roller without resistance. For the next 30 s, that is the entire duration of the test, each participant was asked to express the maximum power that could be delivered while sitting on the saddle, with the roller resistance set to 0.8 Nm·kg^−1^ body mass [6,11,12]. During the test, the researcher provided verbal encouragement to all participants. For statistical analysis, the Mean Power Output (MPO·kg^−1^) and the peak power output (PPO·kg^−1^) during the 30 s were considered.

### Incremental test

After the 30-s Wingate test, each participant rested for 5 minutes at a self-selected pedaling intensity and cadence. Subsequently, an incremental ramp test was performed, starting with a power of 3 W per kg of cyclist’s body mass and augmenting with increments of 1 W·6 s^−1^ until the participant was exhausted [22,23]. The maximum power achieved (MPA·kg^−1^) and the anaerobic threshold power (ATP·kg^−1^) were considered for statistical analysis. The MPA was the highest power maintained for 6 s, which was the last step of the test before failure. The ATP corresponded with the deflection point of the non-linearity of the heart rate response with increasing roller resistance and, thus, power output [24–26].

### Countermovement jump test

The countermovement jump test was performed using the Optojump system (Optojump, Microgate, Bolzano, Italy), consisting of two bars (transmitting and receiving bars, placed 1 m apart) and equipped with 33 optical LEDs fitted in the transmitting bar that continuously communicate with the corresponding set in the receiving bar [27–29].

At the acoustic signal emitted by the software, each participant was instructed to jump as high as possible after completing a countermovement. Each participant performed three trials of jumping with a rest period of 1 minute between jumps to minimize muscular fatigue. All jumps were performed barefoot to negate the effects of shoes, and the hands were placed on the hips to eliminate the contribution of the arms [7]. For statistical analysis, the best jump height (cm) value among the three trials was considered.

### Maximum repetitions test

The participants attended the gym 4 weeks before performing the 1RM test in order to refine the technique of the half squat, deadlift from a standing position, dumbbell lunges, and Bulgarian squat exercises.

For the execution of the 1RM test, we used the protocol carried out in the study by Vikmoen et al. (2016) [30]. First, the participants performed joint mobility exercises of the hips, knees, and ankles for 15 minutes. The 1RM test started with a specific warm-up, consisting of three sets of each exercise with gradually increasing load (40%, 75%, and 85% of the expected 1RM) and decreasing number of repetitions [10,6,3]. The first attempt was performed with a load ~5% below the expected 1RM. The load was increased by ~5% if a lift was successful. The test ended when the participants failed to lift the load in 2–3 attempts, and the highest load successfully lifted was noted as the 1RM. The participants were given 3 minutes of rest between lifts. This procedure was repeated for the 4 exercises (i.e. half squat, deadlift from a standing position, dumbbell lunges, and Bulgarian squat) with 15 minutes of rest between exercises. The 1RM value was used only to define the intensity of the exercises for each participant during the 12 weeks. A pre- and post-comparison of this value was not performed.

### Statistical analysis

Data distribution was evaluated using the Shapiro–Wilk’s test. Data were presented as means ± standard deviations. A post-hoc sample size power analysis using G*Power software 3.1.9.2 (Heinrich Heine University, Düsseldorf, Germany) was calculated (f = 0.40, α = 0.05).

A mixed-factor repeated-measures Analysis of variance (ANOVA) test was used to compare the performance variables over time and between groups. This analysis was performed to assess the interaction effect (i.e. time × group) and the factors main effects (i.e. time and group) on the dependent variables (MPO·kg^−1^, PPO·kg^−1^, MPA·kg^−1^, ATP·kg^−1^, and jump height). In presence of any significant effect, multiple comparisons post hoc test was carried out to analyze the changes in the performance variables. Partial eta-squared was used to assess the effect size (η^2^ = 0.01 indicates a small effect, η^2^ = 0.06 indicates a medium effect, η^2^ = 0.14 indicates a large effect). Statistical significance was set at *P* < .05. All statistical analyses were performed using JAMOVI software package (The jamovi project (2024). jamovi (Version 2.5) [Computer Software]. Retrieved from https://www.jamovi.org).

## Results

Data of all parameters were normally distributed. Means and SD of the data are reported in Table 5.

**Table 5:** Means and SD of the data

	Group A		Group B	
	T0	T1	T0	T1
MPO (W·kg^−1^)	6.42 ± 1.79	8.62 ± 1.26	7.83 ± 2.05	8.74 ± 2.04
PPO (W·kg^−1^)	11.49 ± 2.83	12.32 ± 2.4	12.89 ± 3.6	13.54 ± 2.07
MPA (W·kg^−1^)	3.97 ± 0.51	4.37 ± 0.56	4.54 ± 0.48	4.55 ± 0.57
ATP (W·kg^−1^)	3.64 ± 0.51	3.98 ± 0.48	4.11 ± 0.47	4.08 ± 0.54
Jump height (cm)	23 ± 4.5	24 ± 3.3	30.5 ± 3.8	28.5 ± 5.3

Group A, cyclists who performed strength training in the gym; Group B, cyclists who performed strength training on the bike; MPO, Mean Power Output; PPO, peak power output; MPA, Maximum Power Achieved; ATP, anaerobic threshold power.

Post-hoc power analysis showed that with a sample size of 19 participants, we achieved a power of 91%.

### Results of mixed-factor repeated-measures ANOVA test

#### Wingate test

We found no significant interaction effect in MPO·kg^−1^ values (F_(1,17)_ = 1.99; *P* = .177; ƞ^2^partial = 0.105). There were significant differences in MPO·kg^−1^ values within-subjects over time (F_(1,17)_ = 11.61; *P* = .003; ƞ^2^partial = 0.406). No significant differences in MPO·kg^−1^ values between groups were found (F_(1,17)_ = 1.25; *P* = .279; ƞ^2^partial = 0.068).

No significant differences in PPO·kg^−1^ values were detected in the interaction time × group (F_(1,17)_ = 0.019; *P* = .893; ƞ^2^partial = 0.001), nor within-subjects over time (F_(1,17)_ = 1.34; *P* = .263; ƞ^2^partial = 0.073), nor between groups (F_(1,17)_ = 1.39; *P* = .254; ƞ^2^partial = 0.076).

#### Incremental test

A significant interaction effect in MPA·kg^−1^ was observed (F_(1,17)_ = 6.47; *P* = .021; ƞ^2^partial = 0.276). A significant difference in MPA·kg^−1^ was detected both in within-subjects factor (F_(1,17)_ = 7.62; *P* = .013; ƞ^2^partial = 0.309) and in between groups factor (F_(1,17)_ = 2.51; *P* = .132; ƞ^2^partial = 0.129).

We detected a significant interaction time x group in ATP·kg^−1^ values (F_(1,17)_ = 9.30; *P* = .007; ƞ^2^partial = 0.354) and a main effect in the time factor (F_(1,17)_ = 6.96; *P* = .017; ƞ^2^partial = 0.290). No main effect in the group factor was detected (F_(1,17)_ = 1.68; *P* = .212; ƞ^2^partial = 0.090).

#### Countermovement jump test

We found no significant interaction effect in jump height values (F_(1,17)_ = 3.352; *P* = .085; ƞ^2^partial = 0.165). No main effect within-subjects (F_(1,17)_ = 0.338; *P* = .569; ƞ^2^partial = 0.019) was detected, whereas we identified a main effect between groups (F_(1,17)_ = 11.4; *P* = .004; ƞ^2^partial = 0.400).

### Results of multiple comparisons post hoc test at T0

#### Wingate test

Regarding the MPO·kg^−1^ values, after identifying a significant difference, multiple comparisons post hoc test showed that, at T0, there were no significant differences between groups (*P* = .129).

#### Incremental test

As for the MPA·kg^−1^, since a significant difference was found, multiple comparisons post hoc test revealed that, at T0, there was a significant difference between group A and group B in the MPA·kg^−1^ (*P* = .025) with group B showing higher values than group A.

#### Countermovement jump test

Regarding the jump height, after a significant difference was found, multiple comparisons post hoc test revealed that, at T0, there were significant differences between group A and group B (*P* = .001), with group B showing higher values than group A.

### Results of multiple comparisons post hoc test at T1

#### Wingate test

**Figure 1:**
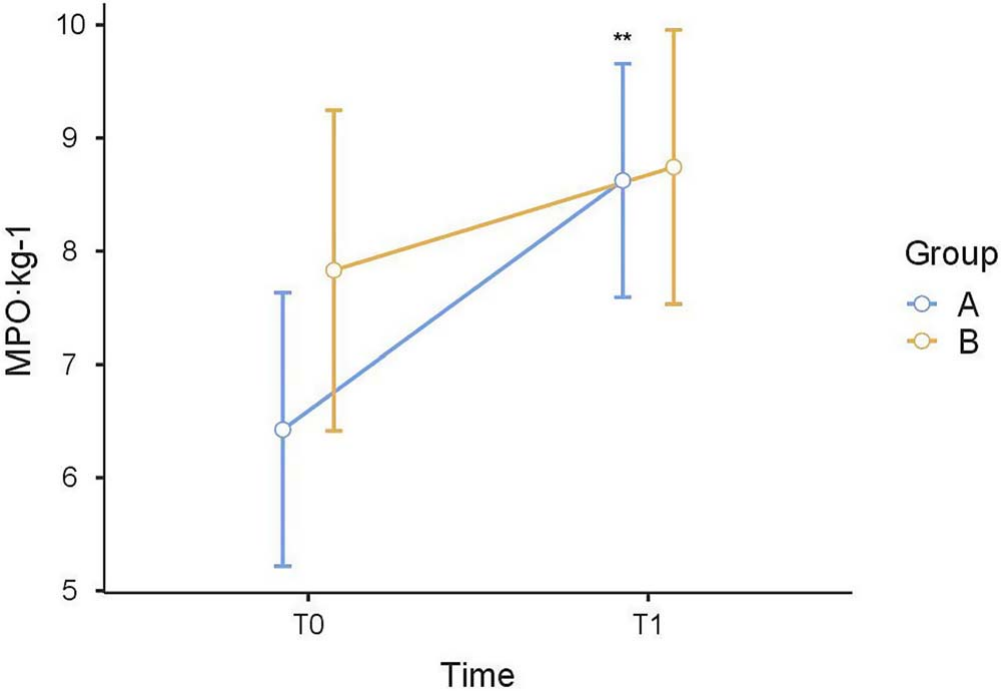
The MPO·kg-1 values of the Wingate test from T0 to T1. *, *P* < .05; **, *P* < .01; ***, *P* < .001.

Regarding the MPO·kg^−1^ values, multiple comparisons post hoc test showed that, at T1, there were no significant differences between group A and group B (*P* = .913). In group A, the MPO·kg^−1^ increased significantly between T0 and T1 (*P* = .002). In group B, no significant differences were found between T0 and T1 (*P* = .276) (Fig. 1).

#### Incremental test

**Figure 2:**
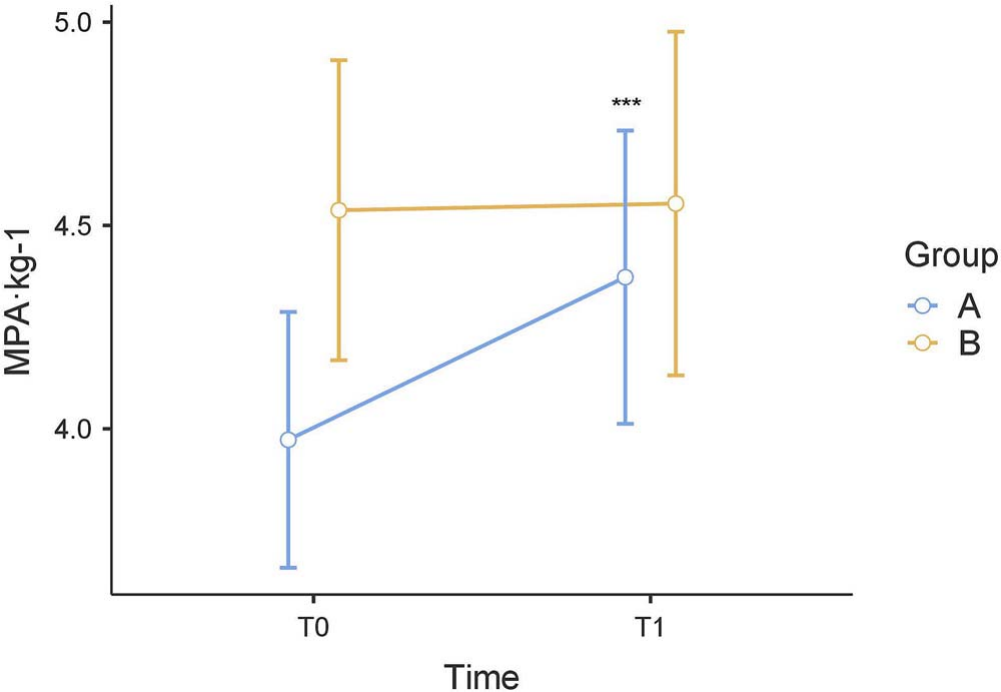
The MPA·kg-1 values of the incremental test from T0 to T1. *, *P* < .05; **, *P* < .01; ***, *P* < .001.

Concerning the MPA·kg^−1^, post hoc multiple comparisons test revealed that there were no significant differences at T1 (*P* = .501) between group A and group B. In group A, the MPA·kg^−1^ increased significantly between T0 and T1 (*P* < .001). In group B, no significant differences were found between T0 and T1 (*P* = .889) (Fig. 2).

**Figure 3:**
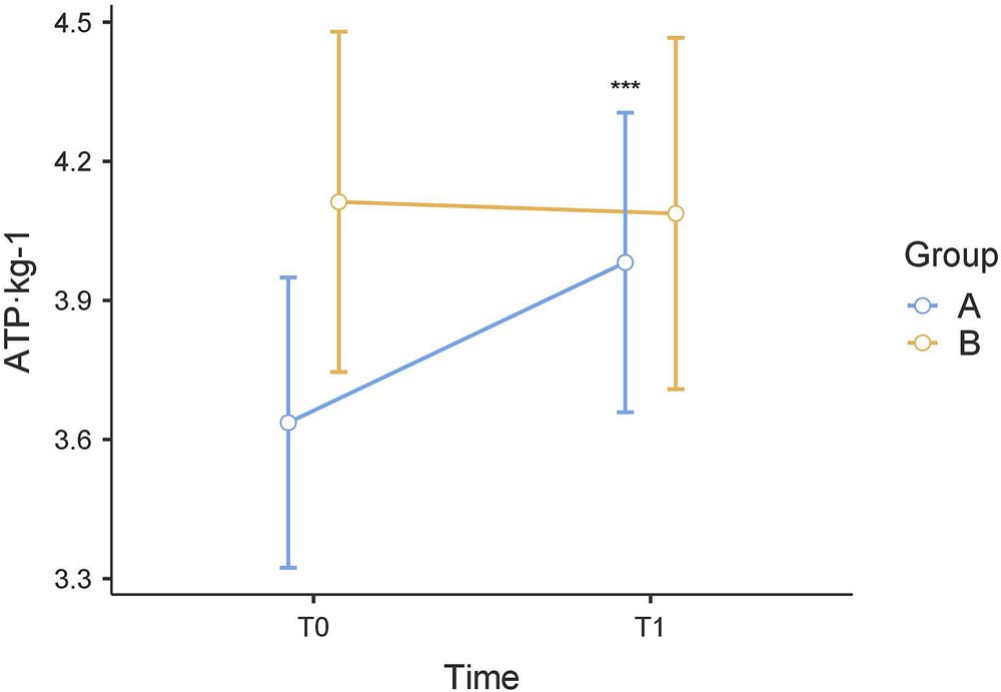
The ATP·kg-1 values of the incremental test from T0 to T1. *, *P* < .05; **, *P* < .01; ***, *P* < .001.

At T1, for the ATP·kg^−1^ values, post hoc multiple comparisons test demonstrated that there were no significant differences between group A and group B (*P* = .660). In group A, the ATP·kg^−1^ increased significantly between T0 and T1 (*P* < .001). In group B, no significant differences were found between T0 and T1 (*P* = .790) (Fig. 3).

#### Countermovement jump test

As regards jump height values, multiple comparison post hoc test showed that there were significant differences between group A and group B at T1 (*P* = .036). In group A, there were no significant differences between T0 and T1 (*P* = .349). In group B, there were no significant differences between T0 and T1 (*P* = .131).

## Discussion

The aim of this study was to compare the effects of 12 weeks of endurance training integrating with strength training performed in the gym (group A) with 12 weeks of endurance training integrating with specific strength training performed on a bike at low pedaling cadences (group B) in young off-road cyclists.

In line with our hypothesis, the group of cyclists who performed endurance training combined with strength training in the gym had significant improvements on some performance indicators compared to the group of cyclists who performed endurance training combined with specific strength training on the bike at low pedaling cadences. In detail, we found improvements in the power output in the Wingate and incremental tests.

The performance indicators we used were the PPO·kg^−1^ and MPO·kg^−1^ for the 30 s Wingate test, the MPA·kg^−1^ and ATP·kg^−1^ for the incremental test, and the jump height in the countermovement jump test.

### Wingate test

At T0, there were no significant differences between the groups in the performance indicators from the 30-s Wingate test. Whereas, in the pre- and post-comparisons, group A showed significant improvements in MPO·kg^−1^ in contrast to group B, which showed no significant differences. As for the PPO·kg^−1^, neither group showed any significant change from T0 to T1.

These results are in line with the study by Rønnestad et al. (2016) previously mentioned [12], which showed that 2 sessions per week for 10 weeks of heavy strength training reported significant improvements in MPO·kg^−1^ [12].

Another study by Rønnestad et al. (2010) aimed to investigate effects of heavy strength training on cycling performance in well-trained cyclists showed that endurance training combined with heavy strength training increase significantly the PPO·kg^−1^ in the 30 s Wingate test [6].

In contrast with our findings, the study by Kristoffersen et al. (2019), who compared the effects of short-sprint training and heavy strength training on endurance performance in well-trained cyclists, showed that after 6 weeks of training only the group performing short-interval training reported significant improvements in MPO·kg^−1^ in the 30 s Wingate test [31]. The fact that the results of this study are in contrast with our findings could be because the program lasted 6 weeks, and therefore, the participants performing strength training probably did not have the same time to adapt as our recruited participants. Furthermore, the participants of this study performed the short intervals at high pedaling cadences (110–120), while in our study, participants performed the intervals with a constrained gear and at a low cadence (40–120). Finally, the participants recruited belonged to different age categories.

The study by Koninckx et al. (2010) compared the effects of a weight training program for the leg extensor with isokinetic cycling training on maximal power output and endurance performance [19]. The result showed that the combination of weight training and endurance training increased maximal power output in the full range of cadences between 40 and 120 rpm while the isokinetic training group increased maximal power between 40 and 100 rpm but not at 120 rpm [19]. Strength training, as part of a cycling endurance training program, is important to consider. Strength training at low pedaling cadences could impair maximal power production at high cadence in elite cyclists. In road and off-road cyclists sprint, pedaling cadence is often in the range of 95–105 rpm. For this reason, high-intensity, low-pedaling-cadence strength training may not be functional for the performance model.

The group B probably did not show improvements in the performance indicators because the cadence they used during the tests (which was unconstrained and thus self-selected) was higher than the strength training exercises they did. They probably improved their strength expression, but only at low cadences, i.e. similar to the strength training exercises they did. In line with the hypothesis, the study by Koninckx et al. (2010) stated that a 12-week isokinetic resistance training protocol at 80 rpm resulted in improved maximal output at or below that cadence but not at higher cadences due to compromised pedaling technique [19].

Furthermore, it is likely that these adaptations could be generated by various factors, such as a higher proportion of type IIA muscle fibers [32]. Probably, as stated in the study by Aagaard et al. (2011), concomitant endurance and strength training do not lead to muscle hypertrophy that otherwise could be observed with heavy-resistance strength training [5].

### Incremental test

Although group A at T0 showed significantly lower values than group B, at T1, group A showed significantly higher values than group B. This result could indicate that the training program performed by group A provided greater improvements on MPA·kg^−1^ and ATP·kg^−1^ of the incremental test. In fact, at T1, only group A showed significant improvements from T0.

In line with our findings, a study by Rønnestad et al. (2015) analyzed the effects of 25 weeks of heavy strength training in young elite cyclists on several indicators of performance, including the MPA in an incremental test [33]. The results of this study showed that concurrent endurance and heavy strength training increased the MPA·kg^−1^ during an incremental test.

A study by Sawyer et al. (2014) showed that 8 weeks of strength training enhanced the peak power achieved during an incremental test [34].

The improvement in MPA·kg^−1^ of the incremental test could be due to the fact that, when reaching the maximum power output, participants used their anaerobic neuromuscular capacities, which are enhanced in the gym and not through specific strength training on the bike. It could also be hypothesized that the overload between strength training on the bike and strength training in the gym was not exactly superimposable.

Also, considering the ATP·kg^−1^, it would appear that strength training performed in the gym could give positive benefits on the increase in ATP.

In agreement with our findings, the study by Rønnestad et al. (2017) [12], investigating the effect of 10 weeks of heavy strength training on cycling performance in elite athletes showed that the group performing strength training in the gym had a tendency toward an improvement on power output at 4 mmol, considered by the scientific literature to be fixed at 4 mmol, the anaerobic threshold [35,36].

The study by Beattie et al (2017) aimed to investigate the effect of a 20-week maximal- and explosive-strength training on cycling performance showed that concurrent bike-specific explosive-strength and endurance training did not improve power at 4 mmol/L [32].

### Countermovement jump test

At T0, the two groups were not homogeneous, as group B showed higher jump height values than group A. In the comparison between T0 and T1, both groups showed no significant differences.

Some studies in the literature have already assessed vertical jump height [12,32].

The results of the study by Beattie et al. (2017) previously mentioned showed that, in line with ours, the change in explosive strength in the intervention group performing bike explosive strength training combined with endurance training was not significantly different from that in the control group performing only endurance training [32].

The study by Rønnestad et al. (2016) showed that a period of 10 weeks of concurrent endurance and heavy strength training significantly improves the squat jump height values [12].

## Conclusion

Cyclists who combine endurance training with strength training in the gym improved several determinants of performance compared to cyclists who performed endurance training with specific strength exercises on a bike at low cadences with a constrained gear. Strength training in the gym combined with endurance training can improve both anaerobic performance and aerobic characteristics.

### Strengths and limitations

The small sample size and the numerical heterogeneity between males and females in the recruited sample represent the main limitations of the study. Nevertheless, the participants belonged to “Esordienti” and “Allievi,” highlighting the homogeneity of the sample in terms of categories. Among the strengths of the study, it must be mentioned the full adherence to strength training on the gym and that there were no dropouts in either group.

### Practical implications

Considering that aerobic and anaerobic performance are the main physiological demands of off-road cycle racing, we suggest that coaches, especially in the transitional period of winter and beyond, include heavy gymnasium strength training sessions for improving and maintaining strength.

## Data Availability

The data underlying this article will be shared on reasonable request to the corresponding author.
